# Supplementary material for: Conserved Pib2 regions have distinct roles in TORC1 regulation at the vacuole
Source: J Cell Sci. 2022 Sep 14;135(18):jcs259994. doi: 10.1242/jcs.259994 (PMC9584352; doi:10.1242/jcs.259994)
Supplement: Supplementary information [file joces-135-259994-s1.pdf]

Supplementary Figure 1.

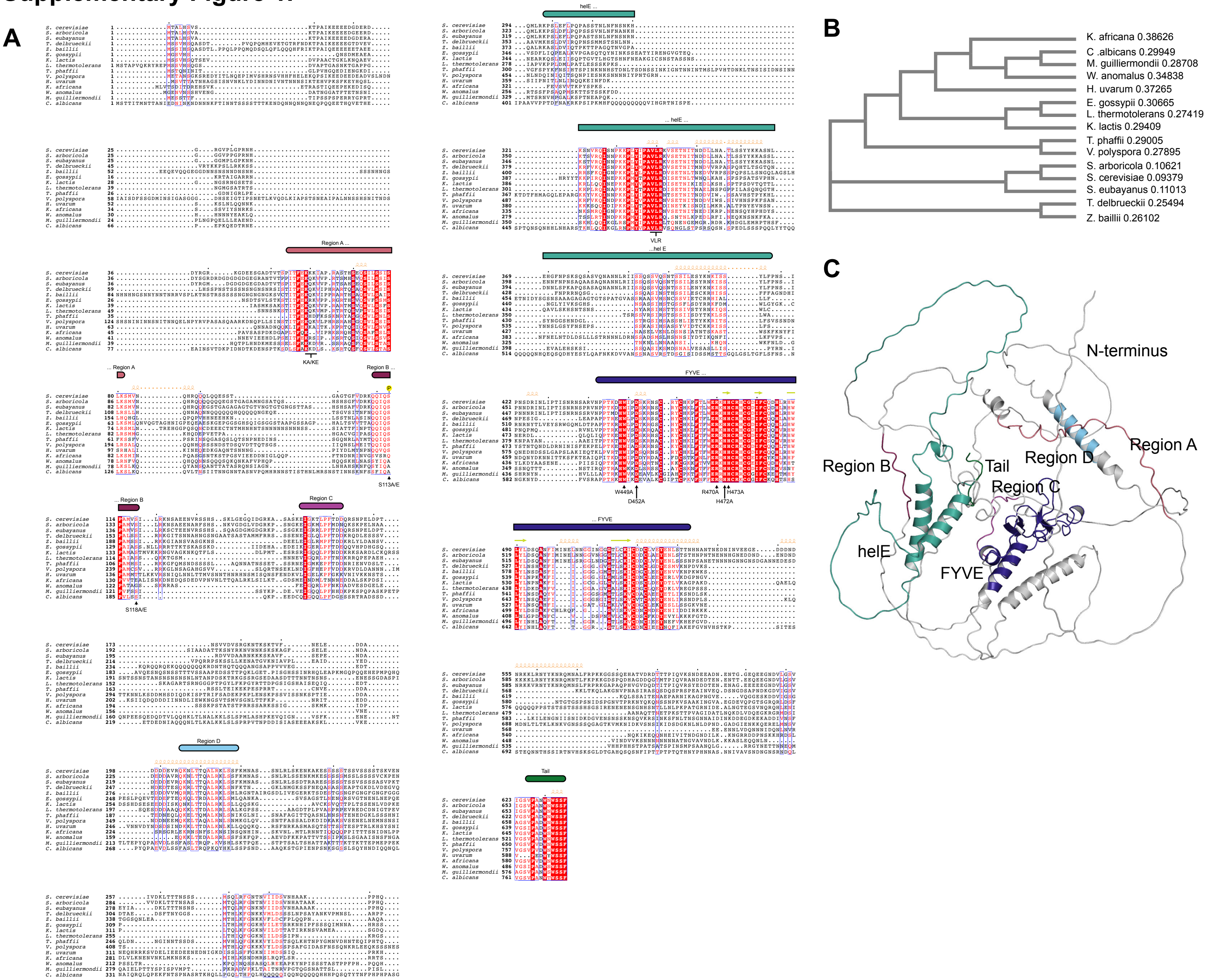

Figure S1. (A) Sequence alignments of Pib2 sequences from ascomycete fungi. Conserved Pib2 regions are labeled as per the *S. cerevisiae* sequence. Identical residues are highlighted in red boxes. Residues with > 70% conservation are colored in red. AlphaFold2 predicted structural elements are indicated above the sequence with alpha-helices represented by orange loops and beta-sheets represented by green arrows. Key conserved residues are shown with arrows below the sequence. Predicted post-translational modifications are shown above the sequence with phosphorylation represented by a P. The sequences used in this alignment are: *Kazachstania Africana* (XP_003958048.1), *Hanseniaspora uvarum* (KKA03925.1), *Candida albicans* (KGU35957.1), *Meyerozyma guilliermondii* (XP_001484326.1), *Wickerhamomyces anomalus* (XP_019038326.1), *Tetrapisispora phaffii* (XP_003687734.1), *Vanderwaltozyma polyspora* (XP_001644984.1), *Eremothecium gossypii* (NP_986037.1), *Kluyveromyces lactis* (XP_452960.1), *Lachancea thermotolerans* (XP_002553804.1), *Saccharomyces arboricola* (EJS43738.1), *Saccharomyces cerevisiae* (NP_011492.3), *Saccharomyces eubayanus* (XP_018222201.1), *Torulaspora delbrueckii* (XP_003682272.1), *Zygosacharomyces bailli* (SJM87752.1). (B) Cladogram of ascomycete species in A generated with sequence alignment. Scores shown to the right of the species name represent the evolutionary sequence distances. (C) AlphaFold2 prediction of structural elements in Pib2 (PDB AF-P53191-F1-model_v1). Conserved regions are colored to match map in Fig. 1A.

Supplementary Figure 2.

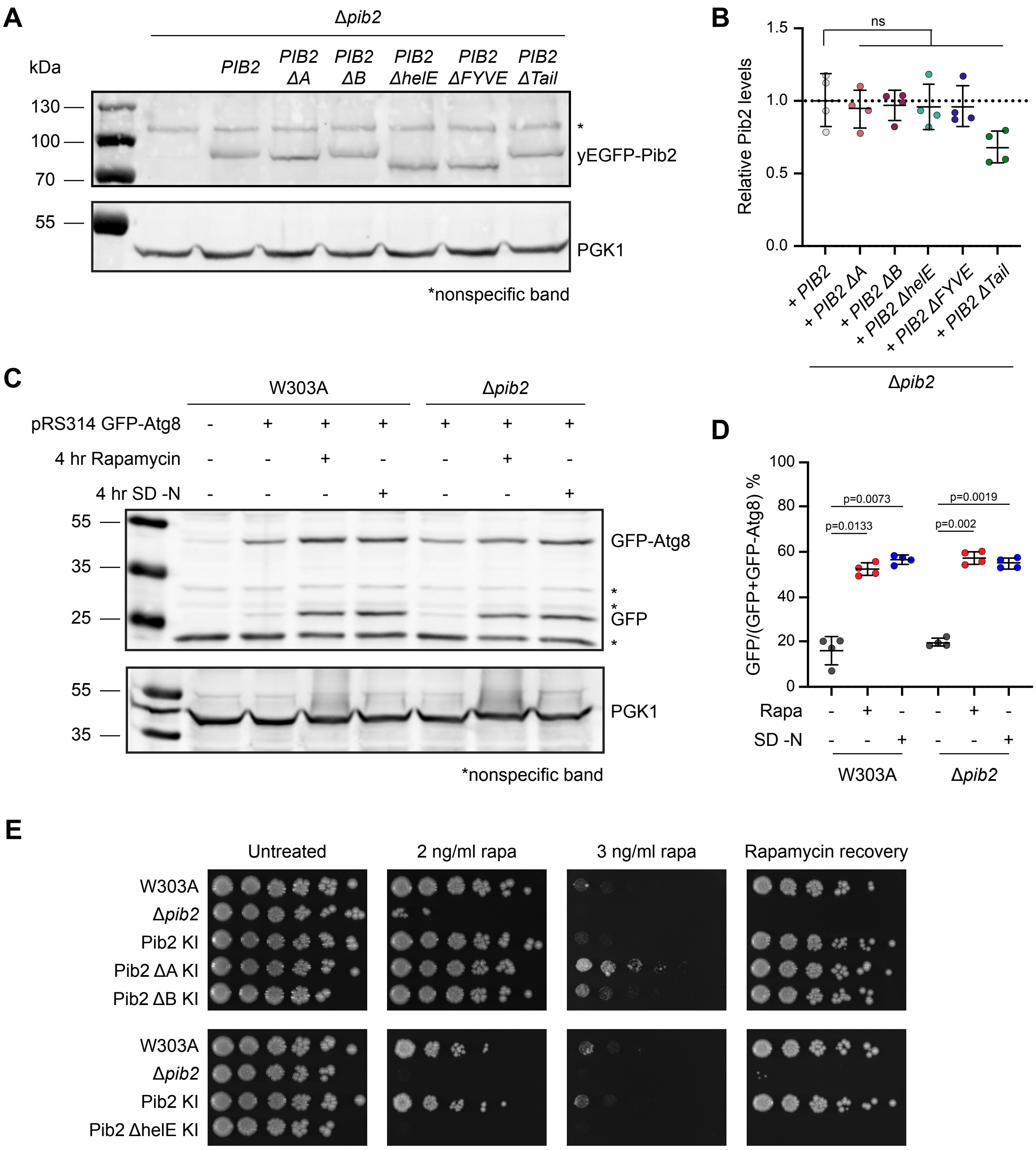

Journal of Cell Science • Supplementary information

Fig. S2. (A) Representative western blot for relative protein expression levels of select yEGFP-Pib2 constructs. (B) Quantification (mean±s.d.) of the blots in A (n=4). Values were normalized to the corresponding Pgk1 loading control. Differences were evaluated by one-way ANOVA ($F = 2.488$, $P = 0.1678$). There were no significant differences from Pib2 expression levels as determined by Tukey multiple comparisons test. (C) Representative western blot assessing induction of autophagy. W303A or Δ*pib2* cells expressing GFP-Atg8 were treated with rapamycin or nitrogen starved for 4 hours. GFP-Atg8 and free GFP were detected using an anti-GFP antibody. (D) Quantification (mean±s.d.) of the blots in C (n=4). Differences in the ratios of free GFP to total GFP for each condition were evaluated by one-way ANOVA ($F = 117.3$, $P < 0.0001$). Significant differences between untreated and treated cells as determined by Tukey post-hoc multiple comparison tests are denoted. No significant differences were observed in comparing W303A and Δ*pib2* cells. (E) Rapamycin exposure and recovery assays of endogenous Pib2 deletion strains performed as described in Figure 1.

Journal of Cell Science • Supplementary information

Supplementary Figure 3.

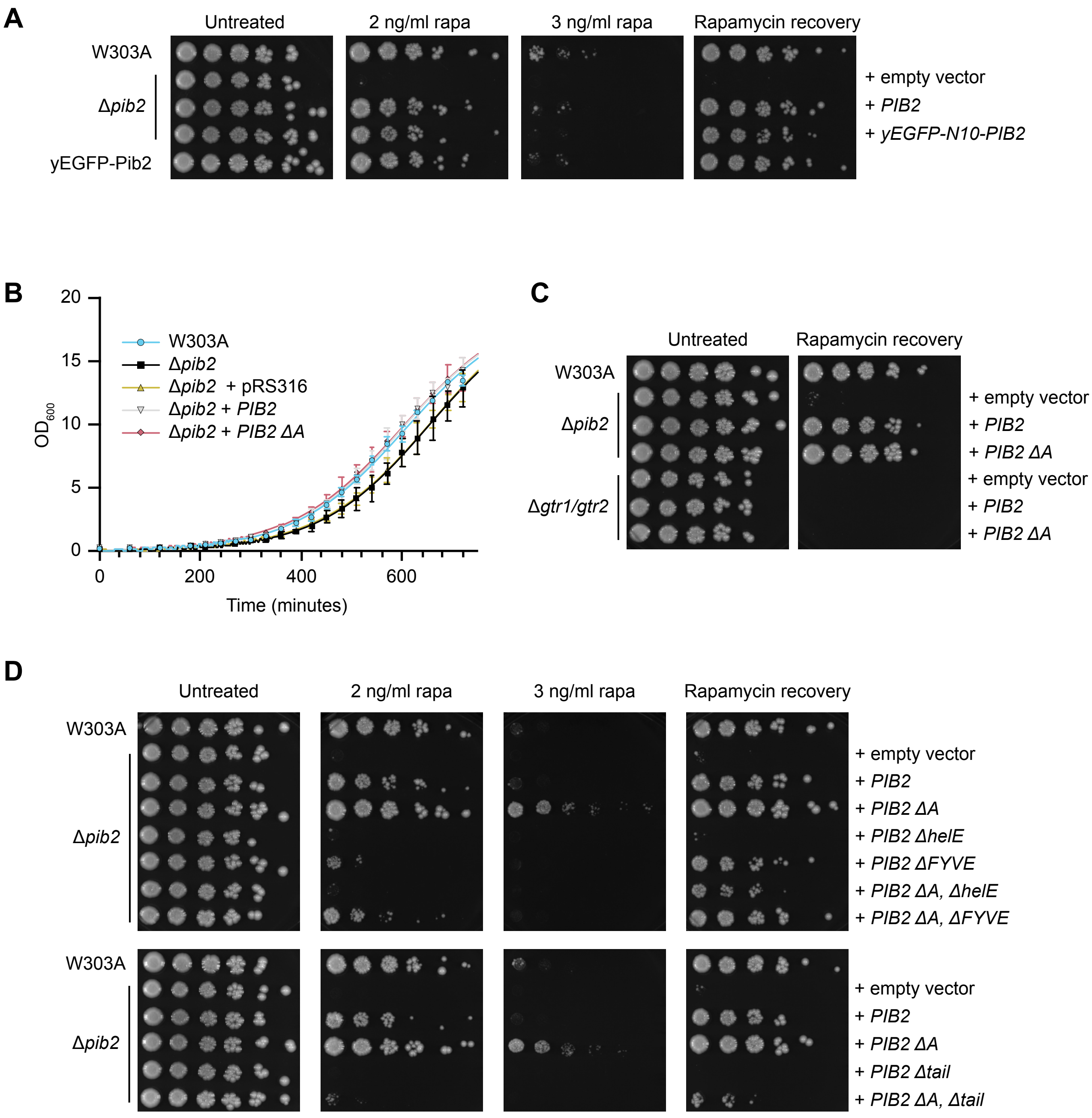

Fig. S3. (A) Rapamycin exposure and recovery assays of an endogenously tagged yEGFP-Pib2 strain performed as in Figure 1. (B) 12-hour growth curve of select yeast strains expressing the indicated Pib2 constructs. (C) Rapamycin exposure and recovery assays of Δ*pib2* or Δ*gtr1/gtr2* cells expressing Pib2 ΔA. Assays performed as in Figure 1, except for rapamycin recovery cells were treated with rapamycin for 2 hours. (D) Rapamycin exposure and recovery assays of Δ*pib2* cells expressing the indicated constructs. These assays were performed as in Figure 1, except for rapamycin recovery cells were treated with rapamycin for 2 hours.

Journal of Cell Science • Supplementary information

Supplementary Figure 4.

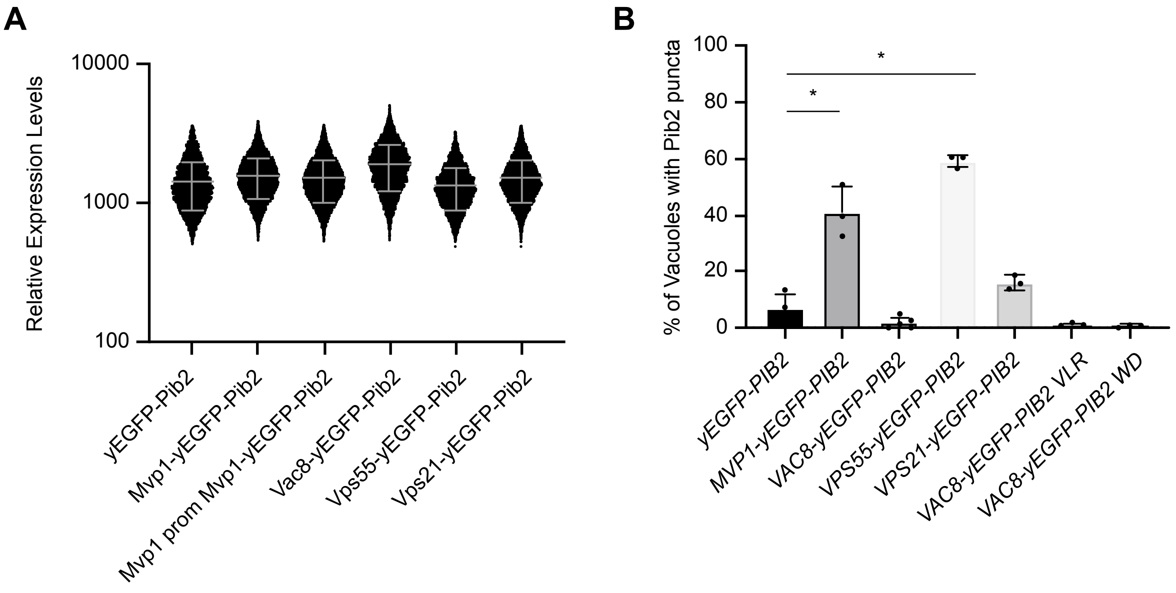

Fig. S4. (A) Quantification (mean±s.d.) of protein expression of targeting constructs based on total GFP fluorescence detected in individual cells normalized to area. 6112-9481 cells were quantified in each case. (B) Quantification (mean±s.d.), from z-stack images, of the percentage of vacuoles containing Pib2 puncta in Δ*pib2* cells expressing the indicated constructs. Differences were assessed by one-way ANOVA ($F = 97.36$, $P < 0.0001$). Significant differences from yEGFP-Pib2 are noted (*$p<0.0001$).

Journal of Cell Science • Supplementary information

Supplementary Figure 5.

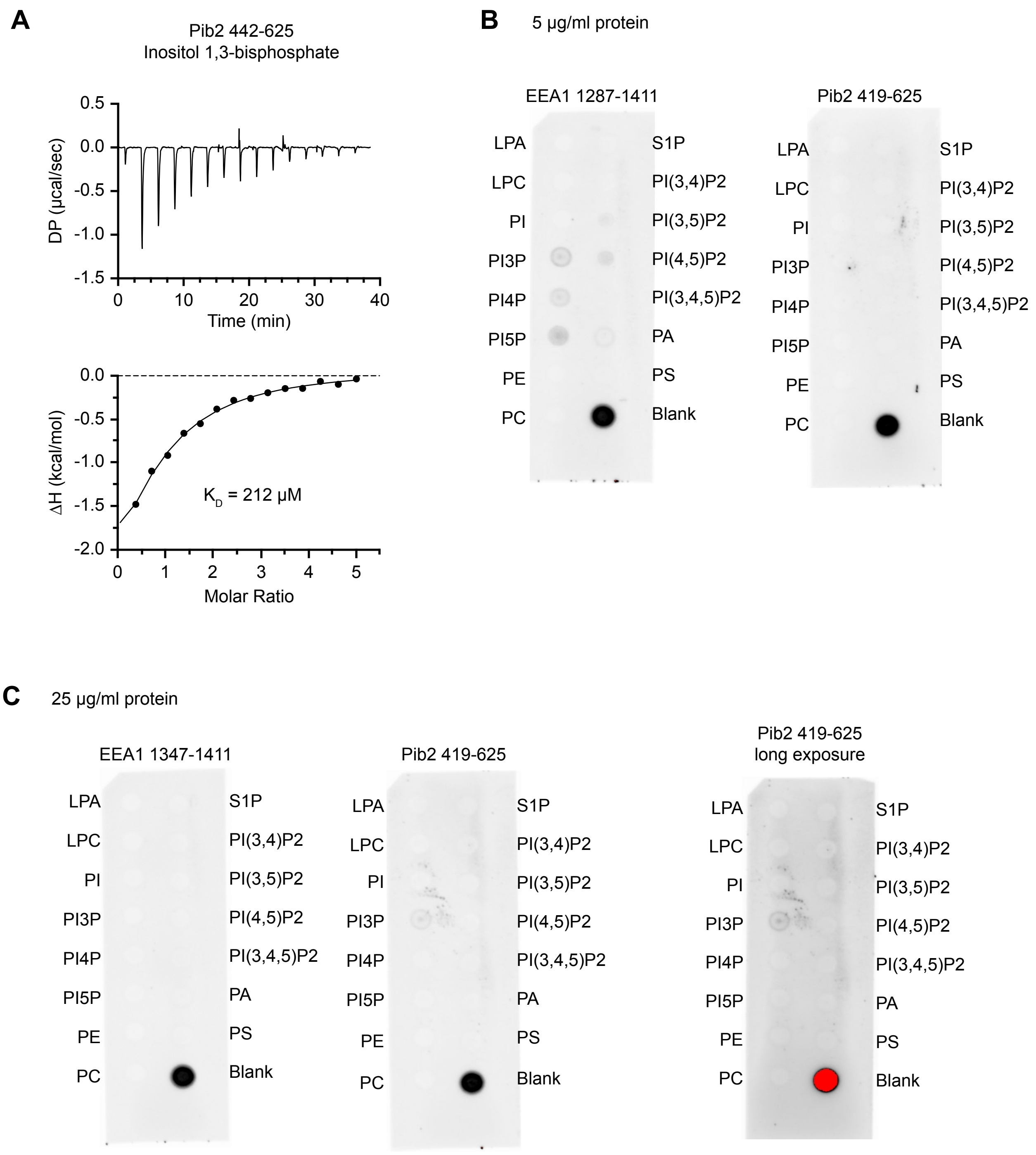

Fig. S5. (A) Binding of inositol 1,3 bisphosphate to S cer. Pib2 442-625 as determined by ITC. The upper panel is a representative thermogram (DP – differential power) and the lower panel is a representative normalized binding isotherm obtained by integration of the peaks shown in the thermogram. The fit is obtained from a single-site binding model (smooth line). The mean determined KD value is shown on the isotherm. (B) Binding of 5 µg/ml of the indicated purified proteins to lipids on a PIP strip. Protein binding determined by anti-His antibody. (C) Binding of 25 µg/ml of the indicated purified proteins to lipids on a PIP strip.

Journal of Cell Science • Supplementary information

Supplementary Figure 6.

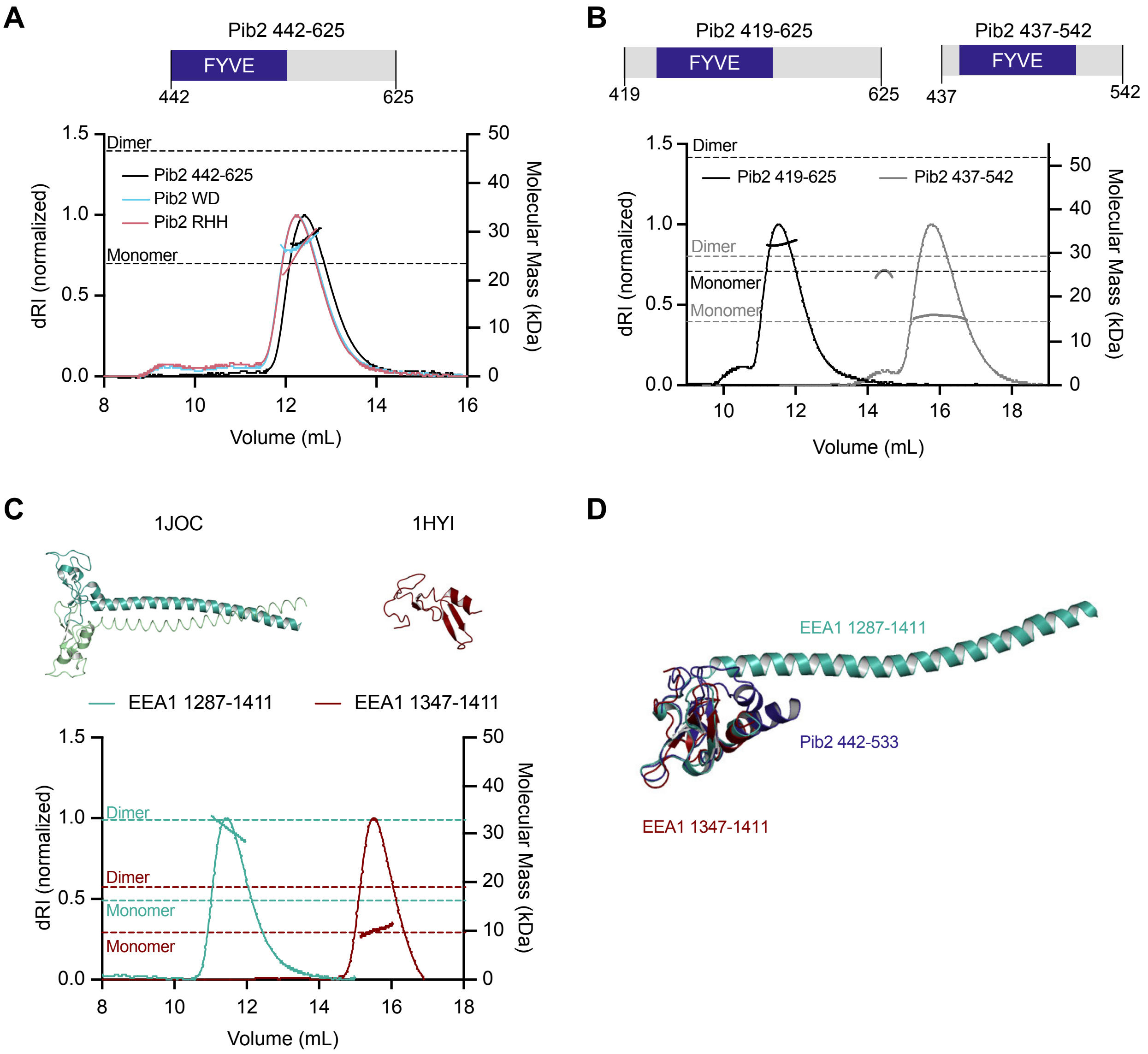

Fig. S6. SEC-MALS (A) Determination of the molar mass of Pib2 442-625 and indicated mutants by SEC-MALS. The theoretical molecular masses of monomers and dimers are shown as dotted lines. (B) Determination of the molar mass of Pib2 419-625 and Pib2 437-542. (C) Determination of the molar mass of two EEA1 FYVE constructs. Boundaries determined from existing structures: EEA1 1287-1411 (PDB: 1JOC) and EEA1 1347-1411 (PDB: 1HYI). (D) Aligned FYVE domains comparing the structures of EEA1 1287-1411 and EEA1 1347-1411 to the AlphaFold2 predicted structure of the Pib2 FYVE domain (residues 442-533). Alignment rendered in PyMOL.

Journal of Cell Science • Supplementary information

Supplementary Figure 7.

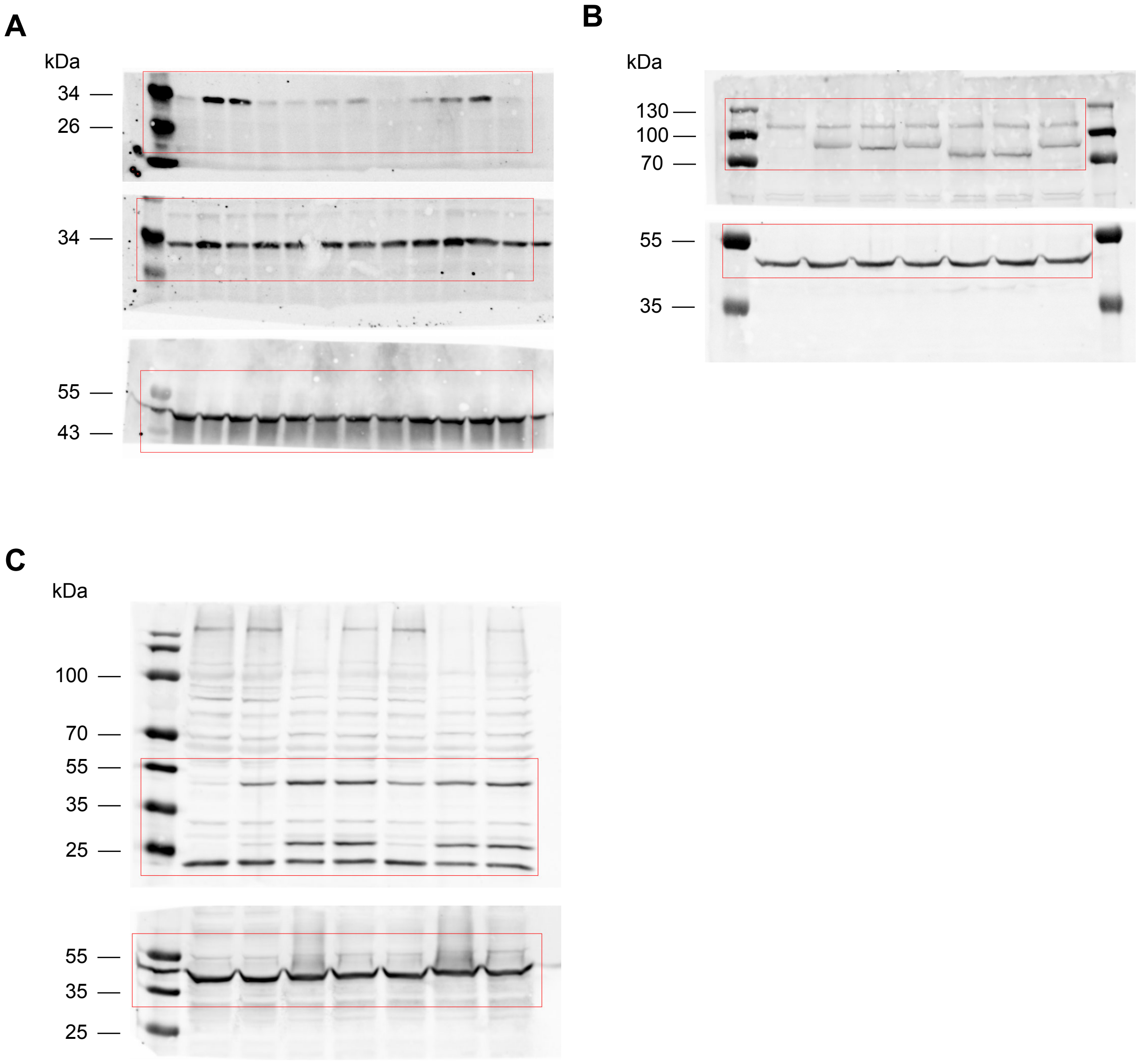

Fig. S7. Blot Transparency Original blots from cropped images in prior figures. Cropped region shown in supplemental figure highlighted by red box. (A) Western blots from Figure 4D. (B) Western blots from Supplemental Figure 2A. (C) Western blots from Supplemental Figure 2C.

Journal of Cell Science • Supplementary information

Table S1. ITC Data

Construct	Cell Concentration (µM)	Syringe Concentration (mM)	K_D (M) ± error of the fit
**Ins(1,3)P2**			
His6-S cer. PIB2 442-625	75	2	51.3E-6 ± 126E-6
His6-S cer. PIB2 442-625	100	4	87.3E-6 ± 48.4E-6
His6-S cer. PIB2 442-625	150	4	178E-6 ± 69.4E-6
His6-S cer. PIB2 442-625	150	4	224E-6 ± 91.7E-6
His6-S cer. PIB2 442-625	150	4	520E-6 ± 355E-6
His6-S cer. PIB2 442-625 R470A, H472A, H473A	100	4	No binding
His6-S cer. PIB2 419-625	200	3	293E-6 ± 402E-6
His6-EEA1 1347-1411	200	3	310E-6 ± 2.02E-3
His6-EEA1 1287-1411	200	3	95.8E-6 ± 53.1E-6
**DiC4 PI3P**			
His6-S cer. PIB2 442-625	150	2	145E-6 ± 271E-6
His6-S cer. PIB2 442-625	150	2	1.03E-3 ± 8.57E-3
His6-S cer. PIB2 442-625	150	2	87.2E-6 ± 35E-6
His6-S cer. PIB2 442-625	150	2	1.55E-3 ± 23.4E-3
His6-S cer. PIB2 442-625	150	2	881E-6 ± 4.32E-3
His6-S cer. PIB2 442-625	153	3	725E-6 ± 809E-6

Journal of Cell Science • Supplementary information

Table S2. Yeast strains used in this work

Strain	Genotype	Reference
W303A	*MAT***a**; *ade2-1*; *leu2-3,112*; *his3-11,15*; *trp1-1*; *ura3-1*; *can1-100*	
W303α	*MAT*α; *ade2-1*; *leu2-3,112*; *his3-11,15*; *trp1-1*; *ura3-1*; *can1-100*	
PY_126	*MAT***a**; *ade2-1*; *leu2-3,112*; *his3-11,15*; *trp1-1*; *ura3-1*; *can1-100*; Δ*pib2*::KAN	Varlakhanova et al., 2017
PY_128	*MAT***a**; *ade2-1*; *leu2-3,112*; *his3-11,15*; *trp1-1*; *ura3-1*; *can1-100*; Δ*pib2*::*HIS3*	This work
PY_104	*MAT***a**; *ade2-1*; *leu2-3,112*; *his3-11,15*; *trp1-1*; *ura3-1*; *can1-100*; Δ*gtr1*::*HIS3*; Δ*gtr2*::KAN	Varlakhanova et al., 2017
PY_262	*MAT***a**; *ade2-1*; *leu2-3,112*; *his3-11,15*; *trp1-1*; *ura3-1*; *can1-100*; yEGFP-N10-*PIB2*::*HIS3*	This work
PY_270	*MAT***a**; *ade2-1*; *leu2-3,112*; *his3-11,15*; *trp1-1*; *ura3-1*; *can1-100*; *PIB2*::KAN	This work
PY_272	*MAT***a**; *ade2-1*; *leu2-3,112*; *his3-11,15*; *trp1-1*; *ura3-1*; *can1-100*; *PIB2* ΔB::KAN	This work
PY_274	*MAT***a**; *ade2-1*; *leu2-3,112*; *his3-11,15*; *trp1-1*; *ura3-1*; *can1-100*; *PIB2* ΔA::KAN	This work
PY_282	*MAT***a**; *ade2-1*; *leu2-3,112*; *his3-11,15*; *trp1-1*; *ura3-1*; *can1-100*; Δ*pib2*::*HIS3;* Δ*vps4*::KAN	This work
PY_284	*MAT***a**; *ade2-1*; *leu2-3,112*; *his3-11,15*; *trp1-1*; *ura3-1*; *can1-100*; *PIB2* ΔhelE::KAN	This work

Journal of Cell Science • Supplementary information

Table S3. Plasmids used in this work

Plasmid		Reference
S cer. PIB2	pRS316 *S cer*. *PIB2* + UTRs	This work
PIB2 ΔA	pRS316 *S cer*. *PIB2* del A (del 54-81) + UTRs	This work
PIB2 ΔB	pRS316 *S cer*. *PIB2* del B (del 109-118) + UTRs	This work
PIB2 ΔC	pRS316 *S cer*. *PIB2* del C (del 152-162) + UTRs	This work
PIB2 ΔD	pRS316 *S cer*. *PIB2* del D (del 205-219) + UTRs	This work
PIB2 ΔhelE	pRS316 *S cer*. *PIB2* del helE (del 298-418) + UTRs	This work
PIB2 ΔFYVE	pRS316 *S cer*. *PIB2* del FYVE (del 442-533) + UTRs	This work
PIB2 ΔTail	pRS316 *S cer*. *PIB2* del Tail (del 626-635) + UTRs	This work
yEGFP-*PIB2*	pRS316 yEGFP-N10-*S cer*. *PIB2* + UTRs	Varlakhanova et al., 2017
yEGFP-*PIB2* ΔA	pRS316 yEGFP-N10-*S cer*. *PIB2* del A (del 54-81) + UTRs	This work
yEGFP-*PIB2* ΔB	pRS316 yEGFP-N10-*S cer*. *PIB2* del B (del 109-118) + UTRs	This work
yEGFP-*PIB2* ΔC	pRS316 yEGFP-N10-*S cer*. *PIB2* del C (del 152-162) + UTRs	This work
yEGFP-*PIB2* ΔD	pRS316 yEGFP-N10-*S cer*. *PIB2* del D (del 205-219) + UTRs	This work
yEGFP-*PIB2* ΔhelE	pRS316 yEGFP-N10-*S cer*. *PIB2* del helE (del 298-418) + UTRs	This work
yEGFP-*PIB2* ΔFYVE	pRS316 yEGFP-N10-*S cer*. *PIB2* del FYVE (del 442-533) + UTRs	This work
yEGFP-*PIB2* ΔTail	pRS316 yEGFP-N10-*S cer*. *PIB2* del Tail (del 626-635) + UTRs	This work
PIB2 VLR	pRS316 *S cer*. *PIB2* 339VLR341->AAA + UTRs	This work
yEGFP-*PIB2* VLR	pRS316 yEGFP-N10-*S cer*. *PIB2* 339VLR341->AAA + UTRs	This work
PIB2 RQI	pRS316 S *cer*. *PIB2* 325RQI327->AAA + UTRs	This work
yEGFP-*PIB2 RQI*	pRS316 yEGFP-N10-S *cer*. *PIB2* 325RQI327->AAA + UTRs	This work
PIB2 PKK	pRS316 S *cer*. *PIB2* 330PKK332->AAA + UTRs	This work
yEGFP-*PIB2 PKK*	pRS316 yEGFP-N10-S *cer*. *PIB2* 330PKK332->AAA + UTRs	This work
PIB2 PLY	pRS316 S *cer*. *PIB2* 333PLY335->AAA + UTRs	This work
yEGFP-*PIB2 PLY*	pRS316 yEGFP-N10-S *cer*. *PIB2* 333PLY335->AAA + UTRs	This work

Journal of Cell Science • Supplementary information

PIB2 WD	pRS316 *S cer*. *PIB2* W449A, D452A + UTRs	This work
yEGFP-*PIB2* WD	pRS316 yEGFP-N10-*S cer*. *PIB2* W449A, D452A + UTRs	This work
PIB2 RHH	pRS316 *S cer*. *PIB2* R470A, H472A, H473A + UTRs	This work
yEGFP-*PIB2* RHH	pRS316 yEGFP-N10-*S cer*. *PIB2* R470A, H472A, H473A + UTRs	This work
yEGFP-*PIB2* ΔhelE ΔFYVE	pRS316 yEGFP-N10-*S cer*. *PIB2* del helE del FYVE + UTRs	This work
PIB2 KA	pRS316 *S cer*. *PIB2* 59KKK61->AAA + UTRs	This work
yEGFP-*PIB2* KA	pRS316 yEGFP-N10-*S cer*. *PIB2* 59KKK61->AAA + UTRs	This work
PIB2 KE	pRS316 *S cer*. *PIB2* 59KKK61->EEE + UTRs	This work
PIB2 KR	pRS316 *S cer*. *PIB2* 59KKK61->RRR + UTRs	This work
PIB2 SA	pRS316 *S cer*. *PIB2* S113A, S118A + UTRs	This work
yEGFP-*PIB2* SA	pRS316 yEGFP-N10-*S cer*. *PIB2* S113A, S118A + UTRs	This work
PIB2 SE	pRS316 *S cer*. *PIB2* S113E, S118E + UTRs	This work
PIB2 KA SA	pRS316 *S cer*. *PIB2* 59KKK61->AAA, S113A, S118A + UTRs	This work
PIB2 KA SE	pRS316 *S cer*. *PIB2* 59KKK61->AAA, S113E, S118E + UTRs	This work
PIB2 KE SA	pRS316 *S cer*. *PIB2* 59KKK61->EEE, S113A, S118A + UTRs	This work
PIB2 KE SE	pRS316 *S cer*. *PIB2* 59KKK61->EEE, S113E, S118E + UTRs	This work
PIB2 ΔA ΔhelE	pRS316 *S cer*. *PIB2* del A del helE + UTRs	This work
PIB2 ΔA ΔFYVE	pRS316 *S cer*. PIB2 del A del FYVE + UTRs	This work
PIB2 ΔA ΔTail	pRS316 *S* cer. *PIB2* del A del Tail + UTRs	This work
VAC8-yEGFP-*PIB2*	pRS316 *VAC8*-yEGFP-N10-*S cer*. *PIB2* + *PIB2* UTRs	This work
MVP1-yEGFP-*PIB2* + *PIB2* UTRs	pRS316 *S cer*. *MVP1*-yEGFP-N10-*S cer*. *PIB2* + *PIB2* UTRs	This work
MVP1-yEGFP-PIB2 + *MVP1* UTRs	pRS316 *S cer*. *MVP1*-yEGFP-N10-*S cer*. *PIB2* + *MVP1* UTRs	This work
VAC8-yEGFP-*PIB2* VLR	pRS316 *VAC8*-yEGFP-N10-*S cer*. *PIB2* 339VLR341->AAA + *PIB2* UTRs	This work
VAC8-yEGFP-*PIB2* WD	pRS316 *VAC8*-yEGFP-N10-*S cer*. *PIB2* W449A, D452A + *PIB2* UTRs	This work
VPS55-yEGFP-*PIB2*	pRS316 *S cer*. *VPS55*-yEGFP-10-*S cer*. *PIB2* + *PIB2* UTRs	This work

Journal of Cell Science • Supplementary information

VPS21-yEGFP-*PIB2*	pRS316 *S cer. VPS21*-yEGFP-10-*S cer. PIB2* + *PIB2* UTRs	This work
PIB2 442-625	pET-15b *S cer*. *PIB2* 442-625	This work
PIB2 437-542	pET-15b *S cer*. *PIB2* 437-542	This work
PIB2 419-625	pET-15b S *cer. PIB2 419-625*	This work
EEA1 1287-1411	pET-15b His6-EEA1(FYVE) 1287-1411	Modified from Addgene #36096 (Burd and Emr, 1998)
EEA1 1347-1411	pET-15b His6-EEA1(FYVE) 1347-1411	Modified from Addgene #36096 (Burd and Emr, 1998)
GFP-*ATG8*	pRS314 GFP-*ATG8*	Modified from Addgene #49425 (Guan et al., 2001)

Journal of Cell Science • Supplementary information
