# Supplementary material for: Conserved Pib2 regions have distinct roles in TORC1 regulation at the vacuole
Source: J Cell Sci. 2022 Sep 14;135(18):jcs259994. doi: 10.1242/jcs.259994 (PMC9584352; doi:10.1242/jcs.259994)
Supplement: Supplementary file 1 [file joces-135-259994_review_history.pdf]

Journal of Cell Science

Conserved Pib2 regions have distinct roles in TORC1 regulation at the vacuole

Kayla K. Troutman, Natalia V. Varlakhanova, Bryan A. Tornabene, Rajesh Ramachandran and Marijn G. J. Ford
DOI: 10.1242/jcs.259994

Editor: Tamotsu Yoshimori

Review timeline

Original submission:	4 March 2022
Editorial decision:	28 March 2022
First revision received:	28 July 2022
Accepted:	15 August 2022

Original submission

First decision letter

MS ID#: JOCES/2022/259994

MS TITLE: Conserved Pib2 regions have distinct roles in TORC1 regulation at the vacuole

AUTHORS: Kayla K Troutman, Natalia V Varlakhanova, Bryan A Tornabene, Rajesh Ramachandran, and Marijn Gerard Johannes Ford
ARTICLE TYPE: Research Article

We have now reached a decision on the above manuscript.

To see the reviewers' reports and a copy of this decision letter, please go to: https://submit-jcs.biologists.org and click on the 'Manuscripts with Decisions' queue in the Author Area. (Corresponding author only has access to reviews.)

As you will see, the reviewers raise a number of substantial criticisms that prevent me from accepting the paper at this stage. However, one feels that a revised version might prove acceptable, if you can address their concerns, while the other reviewer thinks that the work lacks novelty. Then I decided to see a revised manuscript, If you think that you can deal satisfactorily with the criticisms on revision. We would then return it to the reviewers.

Please ensure that you clearly highlight all changes made in the revised manuscript. Please avoid using 'Tracked changes' in Word files as these are lost in PDF conversion.

I should be grateful if you would also provide a point-by-point response detailing how you have dealt with the points raised by the reviewers in the 'Response to Reviewers' box. Please attend to all of the reviewers' comments. If you do not agree with any of their criticisms or suggestions please explain clearly why this is so.

© 2022. Published by The Company of Biologists under the terms of the Creative Commons Attribution License (https://creativecommons.org/licenses/by/4.0/).

Reviewer 1

Advance summary and potential significance to field

The authors investigated how yeast Pib2 protein, an emerging master regulator of TORC1 cell growth controller, works at the molecular level. Via rigorous domain analysis, they demonstrated distinct functions of various conserved regions of Pib2. Their results confirm and extend others' works that took similar approaches.

The highlight of this paper is the functional dissection of endosomal and vacuolar Pib2 pools. This is a fresh approach that none of the preceding papers has taken. This angle is very important and timely, as spatially and functionally distinct pools of TORC1 were recently identified. Their finding of the major role of vacuolar Pib2 (and therefore vacuolar TORC1) in cell growth is important.

I found their experimental data thorough and of high quality. The manuscript is very well written, including methodologies. I therefore support the publication of this fine work in JCS, provided that the following points are addressed.

1) On two occasions, the interpretation of negative results was problematic.
This concern should be addressed by adding proper positive controls (see major points).

2) A significant part of domain analysis overlaps with previous reports (e.g., Kim and Cunningham 2015, Michel et al 2017, Ukai et al 2018, and Tanigawa et al 2021). Although these preceding works were properly acknowledged in most cases there are a few occasions that warrant additional mentions (see minor points).

Comments for the author

Major points:
1. In Figure 4, the loss-of-function of endosomal Pib2 constructs might not be due to their localization; the addition of endosomal targeting domains (Mvp1 or Vps55) might have simply abandoned the functionality of Pib2 by whatever means.
This is particularly concerning for Mvp1, because its mutant type, which allowed normal Pib2 localization, yet remained unfunctional. To ensure their biochemical functionality, authors should demonstrate that these constructs can properly activate the endosomal TORC1 pool (various readouts are available; see Hatakeyama et al 2019 and Chen et al 2021).
2. In Figure S5, the experimental setting for the FYVE-PI3P head binding is quite different from in vivo situations, in which PI3P is embedded within membranes. It is therefore important to demonstrate that the assay itself is properly working, by adding a positive control (=another PI3P-binding domain that shows a higher affinity).

Minor points (needs text editions only):
L48. It is misleading to include Rps6 here, as Rps6 is an indirect downstream factor, while others (Sch9, Atg13 and Vps27) are all direct substrates of TORC1.
L54. Cite PMID: 30732525, in which signaling endosomes were first defined.
L62-63. EGO stands for "exit (not escape) from rapamycin-induced growth arrest".
Here, Citing Dubouloz et all 2005 is most appropriate, as this paper defined EGO.
L75 and L80. Spell out PI3P and PI(3,5)P2 in full at their first appearance.
L76-79. Not sure if mTORC1 regulation by Vps34 is mediated by Rags. PMID:
27477288 rather proposes "Vps34-PLD and Rag functioning in parallel to regulate mTORC1 activation".
L153-154. Discuss this conclusion (key roles of regions A and B) in comparison with the work by Ukai et al (2018), which showed a critical role of 1-50aa.
L190-194. Mention that the partial (but not complete) delocalization of deltaFYVE agrees with Ukai et al, (2018).
L194-195. As the E region is a TORC1 binding domain, the Pib2 recruiting factors in the "dual recruitment mechanism" can well be TORC1 and PI3P. This possibility is worth being pointed out.
L203-206. Mention that this conclusion agrees with Hatakeyama et al (2019 Mol Cell).
L260-261. Mention that this conclusion agrees with Michel et al (2017).

© 2022. Published by The Company of Biologists under the terms of the Creative Commons Attribution License (https://creativecommons.org/licenses/by/4.0/).

L284-287. Does this result suggest that Ypk3, the kinase for Rps6, a substrate of vacuolar (rather than endosomal) TORC1? Please discuss. Also, cite PMID: 26582391, which demonstrated Ypk3 as a TORC1 substrate.
L284-287. Also, discuss kinetics; Vac8-Pib2 seemed to take longer than wild-type to activate TORC1. Cite PMID: 25063813 in relation to this argument.
L484-485. The essential role of vacuolar Pib2 (and thus vacuolar TORC1) in cell growth is consistent with the fact that vacuolar TORC1 targets Sch9 (Hatakeyama et al 2019), which is very important for cell growth (Urban et al, 2007). This point should be discussed.
Figure 6. Say “Signaling endosome” rather than “Endosome”.
Figure 6. In the enlarged cartoon, omit “autophagy”, as there is no evidence that the vacuolar TORC1 pool regulates autophagy.
Figure S4C and D. This data is important as a biochemical readout of TORC1 activity (even though indirect). I suggest moving this to the main Figure 4.

Reviewer 2

Advance summary and potential significance to field

The manuscript entitled “Conserved Pib2 regions have distinct roles in TORC1 regulation at the vacuole” by Troutman et al., describes about regulatory mechanism of Pib2, which regulates TORC1, by making mutant and chimera proteins. The authors narrowed down the amino acids residues in Pib2 that suppressively functions in TORC1 regulation. They also found that FYVE domain is not necessary for recovery from rapamycin exposure. In addition, they described that endosomal localization of Pib2 is not needed for this process.

Comments for the author

In general, the data are mostly convincing and the experiments were moderately performed. However, regrettably, this study lacks enthusiastic novelty that warrant the publication in JCS. Most of novel points described in this study is detailed analysis of the preceding works but not leading to our deepened understanding of the mechanisms.
Specific points 1 Figure 1 C. The authors concluded that rapamycin recovery is defected in pib2 null and several mutants based on plating assay. Does this mean that the cells are irreversibly dead? The authors need to support this point from the other line of evidence. What does rapamycin recovery means in mechanistic context. Without it, it is not enough for this journal.
2 Figure 3. It deserved to be reported that specific amino acids necessary for TORC1 suppression. However the analysis were shallow for this journal. For example, does S to A mutant actually affect the phosphorylation status of Pib2?n Line 234 How about K to R mutant?
3 Figure 6B The picture is missing superscript.
4 The conclusion obtained from chimera proteins are mostly similar to that of Hatakeyama paper.

First revision

Author response to reviewers' comments

Reviewer 1 Advance Summary and Potential Significance to Field:
The authors investigated how yeast Pib2 protein, an emerging master regulator of TORC1 cell growth controller, works at the molecular level. Via rigorous domain analysis, they demonstrated distinct functions of various conserved regions of Pib2. Their results confirm and extend others’ works that took similar approaches.

The highlight of this paper is the functional dissection of endosomal and vacuolar Pib2 pools. This is a fresh approach that none of the preceding papers has taken. This angle is very important and timely, as spatially and functionally distinct pools of TORC1 were recently identified. Their finding of the major role of vacuolar Pib2 (and therefore vacuolar TORC1) in cell growth is important.

© 2022. Published by The Company of Biologists under the terms of the Creative Commons Attribution License (https://creativecommons.org/licenses/by/4.0/).

I found their experimental data thorough and of high quality. The manuscript is very well written, including methodologies. I therefore support the publication of this fine work in JCS, provided that the following points are addressed.

1) On two occasions, the interpretation of negative results was problematic.
This concern should be addressed by adding proper positive controls (see major points).

2) A significant part of domain analysis overlaps with previous reports (e.g., Kim and Cunningham 2015, Michel et al 2017, Ukai et al 2018, and Tanigawa et al 2021). Although these preceding works were properly acknowledged in most cases, there are a few occasions that warrant additional mentions (see minor points).

We thank the reviewer for their positive comments and helpful suggestions.

Reviewer 1 Comments for the Author:

Major points:
1. In Figure 4, the loss-of-function of endosomal Pib2 constructs might not be due to their localization; the addition of endosomal targeting domains (Mvp1 or Vps55) might have simply abandoned the functionality of Pib2 by whatever means. This is particularly concerning for Mvp1, because its mutant type, which allowed normal Pib2 localization, yet remained unfunctional. To ensure their biochemical functionality, authors should demonstrate that these constructs can properly activate the endosomal TORC1 pool (various readouts are available; see Hatakeyama et al 2019 and Chen et al 2021).

We thank the reviewer for this suggestion. The reviewer's concern is appreciated. We fully agree that showing the endosomal effects of Pib2 would be the most direct way to address the concern about the activity of the Mvp1-yEGFP-Pib2 constructs. We would point out that it is currently unknown if Pib2 is necessary for activation of TORC1 in the endosomal branch, which has been shown to control autophagy (Hatakeyama et al., 2019). Indeed, in Figure S2C-D, we show that **in Δ*pib2* cells, autophagy induction is indistinguishable from control W303A cells**. This has also been shown previously (Varlakhanova et al., 2017). Therefore, there is no known readout for the role of Pib2 in regulation of TORC1 localized at the endosome.

The reviewer's concern arises from our experiments with Mvp1-yEGFP-Pib2 and Mvp1 mut1- yEGFP-Pib2 (see below). These were our first constructs attempting to localize Pib2 to the endosome. For Mvp1-yEGFP-Pib2, the localization was indeed endosomal, as expected, and this construct did not support growth after rapamycin exposure. To validate this, we then generated Mvp1 mut1-yEGFP-Pib2. Mvp1 mut1 is PI3P-binding-deficient (Sun et al., 2020) and, when expressed in Δ*mvp1* cells, Mvp1 mut1 is cytosolic. However, this chimeric construct had a broadly vacuolar, not cytosolic, distribution, but this was not homogeneous and appeared patchy. It also did not support recovery after rapamycin exposure. One caveat for this localization is that the cells used still contain endogenous Mvp1, which may dimerize with the Mvp1 mut1-containing construct, as would be predicted by the structure and known Mvp1 functions. Therefore, localization of Mvp1 mut1-yEGFP-Pib2 and Pib2 function in this context may be confounded.

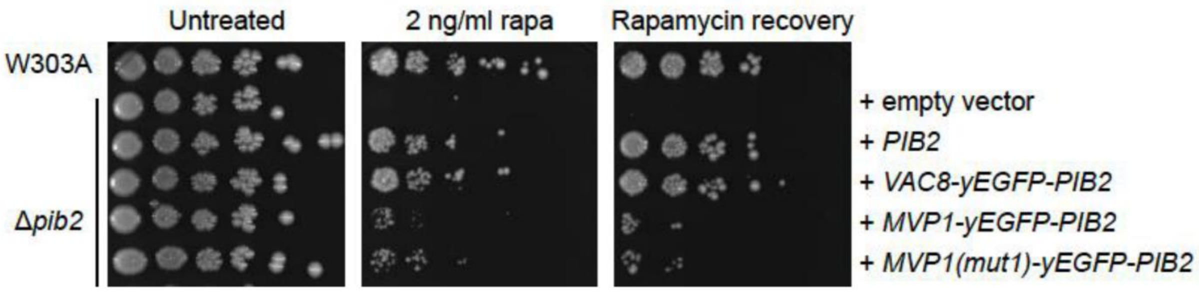

Therefore, to address the functionality of Pib2 in these Mvp1 constructs, we made two additional constructs: Vac8-Mvp1-yEGFP-Pib2 and Vac8-Mvp1 mut1-yEGFP-Pib2. We reasoned that if Mvp1-yEGFP-Pib2 is coupled with a vacuole-targeted protein (Vac8), it may override or counteract some

© 2022. Published by The Company of Biologists under the terms of the Creative Commons Attribution License (https://creativecommons.org/licenses/by/4.0/).

of the targeting introduced by Mvp1 or Mvp1 mut1, allowing us to assess whether Pib2 remains functional. These constructs had similar subcellular distributions to WT Pib2, with a large vacuolar component and some puncta (see below). We find, as shown below, that Vac8- Mvp1 mut1-yEGFP-Pib2 is significantly better at supporting rapamycin recovery than is Vac8- Mvp1-yEGFP-Pib2, where Mvp1 is wild type (below). However, in both cases, growth is slower than that observed in W303A cells or Δ*pib2* cells expressing Pib2, perhaps because of continued influence of the Mvp1 or Mvp1 mut1, and possible influence of endogenous Mvp1. Nevertheless, relocalization of Pib2 to the vacuole does permit partial recovery and hence Pib2 in this context is not compromised. Because of the confounding localizations associated with Mvp1 mut1, we introduced also Vps55, which lacks these concerns.

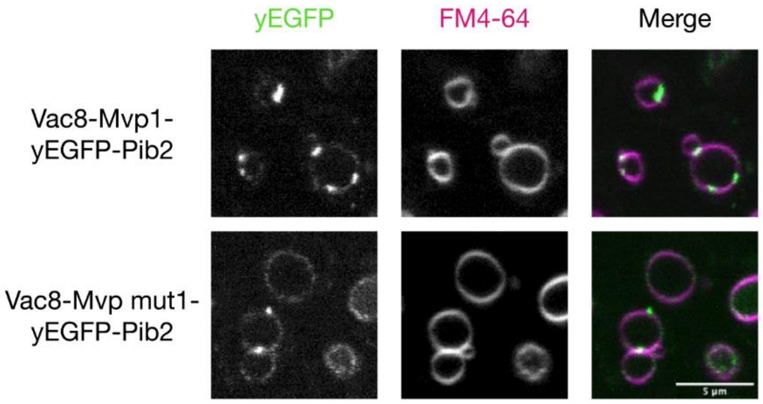

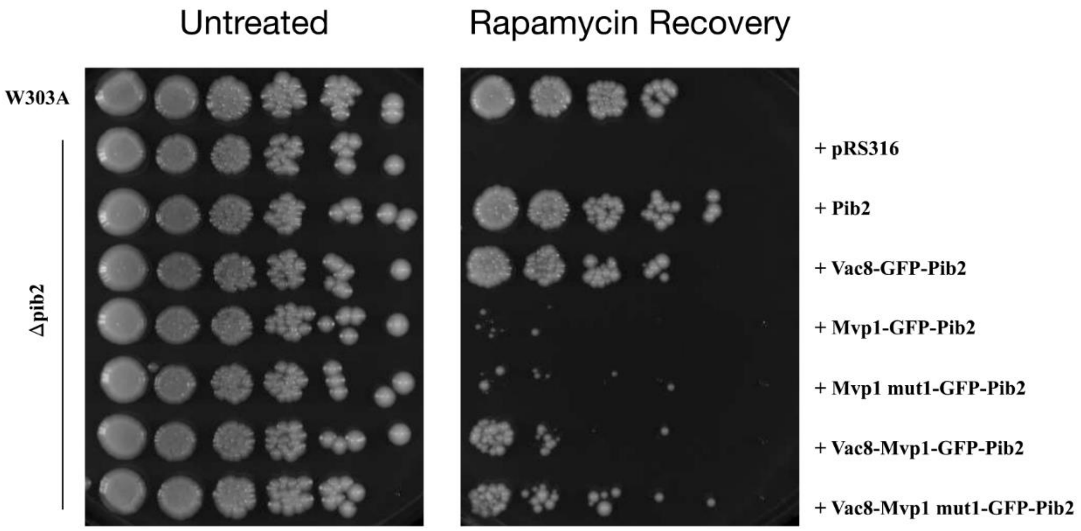

We made Vps55-GFP and compared its distribution to that of Vps55-yEGFP-Pib2. Both Vps55- yEGFP and Vps55-yEGFP-Pib2 had similar distributions in the cell (see below and Fig. 4A). Vps55 is membrane anchored and here its distribution is “overriding” Pib2’s endogenous targeting mechanisms. This construct cannot reactivate growth following rapamycin exposure.

© 2022. Published by The Company of Biologists under the terms of the Creative Commons Attribution License (https://creativecommons.org/licenses/by/4.0/).

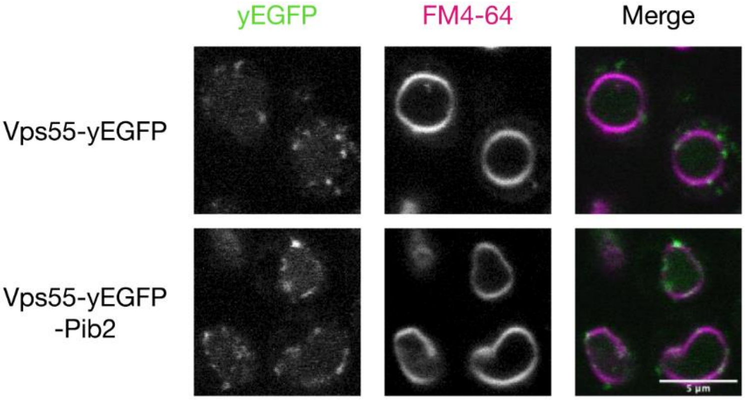

We also generated Vps21-yEGFP-Pib2. Vps21 was a deliberate choice to test the effects of a non-functional protein fused to the N-terminus of yEGFP-Pib2: addition of yEGFP-Pib2 to the C-terminus of Vps21 affects its lipidation that occurs to promote its membrane association (UniProt P36017). Indeed, and as expected, Vps21-yEGFP had a cytosolic distribution (see figure below), whereas Vps21-yEGFP-Pib2 was largely vacuolar, with a distribution similar to what we observe with yEGFP-Pib2 (see below and Fig. 4A). This construct therefore allowed Pib2 to direct subcellular localization and rapamycin assays showed that addition of Vps21 did not affect Pib2's ability to reactivate Pib2 (Fig. 4C), supporting recovery from rapamycin indistinguishable from yEGFP-Pib2. Hence, the presence of a nonfunctional protein does not impede Pib2 activity and Pib2 is functional in this context.

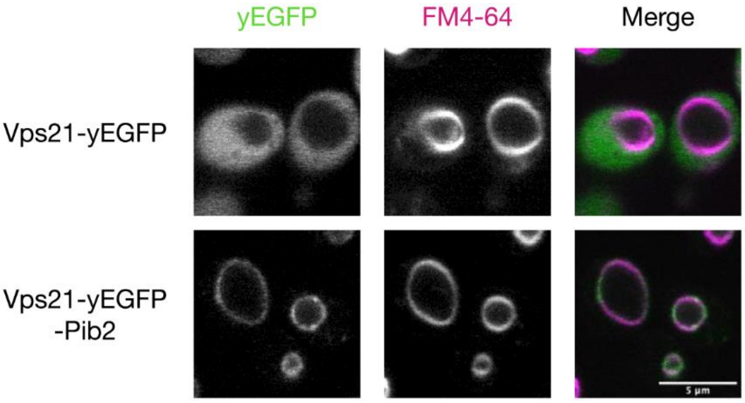

We have also demonstrated that N-terminally tagging Pib2 with yEGFP alone does not affect its function. We carefully compared both plasmid- and genomically-expressed yEGFP-Pib2 with non-tagged Pib2 and observed that the tagging did not have an effect on Pib2's ability to reactivate TORC1 (Fig. S3A). This has also been shown previously by others (Ukai et al., 2018). Furthermore, the AlphaFold prediction of Pib2 shows that, consistent with other bioinformatic analyses, it is largely unstructured. Tagging is therefore unlikely to affect function by localized compromising of a Pib2 fold.

Lastly, of note, we incorporated Pib2 mutations into the Vac8-yEGFP-Pib2 construct to show that vacuolar localization is not sufficient for TORC1 reactivation. The two constructs we used are Vac8-yEGFP-Pib2 WD (FYVE domain mutation) and Vac8-yEGFP-Pib2 VLR (helE mutation). Using Vac8-yEGFP-Pib2 WD to bring this mutant to the vacuole rescued the localization and growth defects of the Pib2 WD mutant. Conversely, the Vac8-yEGFP-Pib2 VLR mutant was still unable to rescue growth following rapamycin exposure. This suggests interactions with TORC1 mediated by the Pib2 helE region are necessary for this interaction. See Figure 6.

Because of the complications that may arise with the endogenous Mvp1, we have removed the experiments with Mvp1 Mut1 from the revised manuscript.

2. In Figure S5, the experimental setting for the FYVE-PI3P head binding is quite different from in vivo situations, in which PI3P is embedded within membranes. It is therefore important to

© 2022. Published by The Company of Biologists under the terms of the Creative Commons Attribution License (https://creativecommons.org/licenses/by/4.0/).

demonstrate that the assay itself is properly working, by adding a positive control (=another PI3P-binding domain that shows a higher affinity).

We thank the reviewer for this helpful comment. We now provide the following controls.

We generated several additional FYVE domain constructs. We made a longer version of Pib2 – Pib2 419-625 – as well as two versions of the EEA1 FYVE domain. EEA1 is a canonical and thoroughly characterized FYVE domain so we used this as a positive control for a PI3P-binding FYVE domain (Burd and Emr, 1999). The boundaries of the EEA1 FYVE domain constructs we used were determined from the boundaries of the existing EEA1 FYVE constructs: PDB ID 1JOC (1287-1411) and 1HYI (1347-1411).

EEA1 1287-1411 is dimeric as demonstrated using SEC-MALS and had a slightly higher affinity for the PI3P head group. While still in the micromolar range, this is comparable to previously published affinities (Gaullier et al., 2000). Our new data are presented in updated Figures S5 and S6 and Table S1.

EEA1 1347-1411 was largely monomeric as demonstrated using SEC-MALS. This is consistent with its NMR structure and can be rationalized as these constructs lack the dimerizing N-terminal helix that is found in EEA1 1287-1411. ITC experiments with this construct showed binding affinities similar to that of our Pib2 FYVE domain constructs. See Figures S5 and S6 and Table S1.

Each of the Pib2 FYVE domains were monomeric (S6A-B), perhaps because they lack a predicted N-terminal helix as seen in EEA1 1287-1411. This may be why our observed affinities for headgroup are similar to that for monomeric EEA1 FYVE.

Minor points (needs text editions only):
L48. It is misleading to include Rps6 here, as Rps6 is an indirect downstream factor, while others (Sch9, Atg13 and Vps27) are all direct substrates of TORC1.
We agree and have rearranged the text accordingly. ~L55

L54. Cite PMID: 30732525, in which signaling endosomes were first defined.
This citation was added. ~L61

L62-63. EGO stands for “exit (not escape) from rapamycin-induced growth arrest”. Here, Citing Dubouloz et all 2005 is most appropriate, as this paper defined EGO.
We apologize for this nomenclature error. We have fixed this and added the appropriate citation. ~L69-70

L75 and L80. Spell out PI3P and PI(3,5)P2 in full at their first appearance.
This was addressed. ~L81 & L87

L76-79. Not sure if mTORC1 regulation by Vps34 is mediated by Rags. PMID: 27477288 rather proposes “Vps34-PLD and Rag functioning in parallel to regulate mTORC1 activation”.
We have reworded the text here and have added the appropriate citation. ~L83-86

L153-154. Discuss this conclusion (key roles of regions A and B) in comparison with the work by Ukai et al (2018), which showed a critical role of 1-50aa.
This was addressed in section “N-terminal Pib2 regions A and B display a TORC1 inhibitory function” (~L223) and in the discussion (~L495-500).

L190-194. Mention that the partial (but not complete) delocalization of deltaFYVE agrees with Ukai et al, (2018).
This is addressed ~L201, L551.

L194-195. As the E region is a TORC1 binding domain, the Pib2 recruiting factors in the “dual recruitment mechanism” can well be TORC1 and PI3P. This possibility is worth being pointed out.
This is addressed ~L555-558.

© 2022. Published by The Company of Biologists under the terms of the Creative Commons Attribution License (https://creativecommons.org/licenses/by/4.0/).

L203-206. Mention that this conclusion agrees with Hatakeyama et al (2019 Mol Cell).
This was added ~L216-218.

L260-261. Mention that this conclusion agrees with Michel et al (2017).
This was added ~L275-276.

L284-287. Does this result suggest that Ypk3, the kinase for Rps6, a substrate of vacuolar (rather than endosomal) TORC1? Please discuss. Also, cite PMID: 26582391, which demonstrated Ypk3 as a TORC1 substrate.
This result could suggest that Ypk3 is a substrate of vacuolar TORC1. This was discussed ~L308-311

L284-287. Also, discuss kinetics; Vac8-Pib2 seemed to take longer than wild-type to activate TORC1. Cite PMID: 25063813 in relation to this argument.
Vac8-Pib2 does appear to take longer to activate TORC1, this could be due to the lack of a rapid response as described by Stracka et al., 2014. This is addressed in the text ~L303-308.

L484-485. The essential role of vacuolar Pib2 (and thus vacuolar TORC1) in cell growth is consistent with the fact that vacuolar TORC1 targets Sch9 (Hatakeyama et al 2019), which is very important for cell growth (Urban et al, 2007). This point should be discussed.
We have raised this point in our revised discussion. ~L565-567

Figure 6. Say "Signaling endosome" rather than "Endosome".
Figure 7 was updated to reflect this distinction.

Figure 6. In the enlarged cartoon, omit "autophagy", as there is no evidence that the vacuolar TORC1 pool regulates autophagy.
This has been updated in Figure 7.

Figure S4C and D. This data is important as a biochemical readout of TORC1 activity (even though indirect). I suggest moving this to the main Figure 4.
We thank the reviewer for this suggestion and have moved the glutamine response panels to the main Figure 4.

Reviewer 2 Advance Summary and Potential Significance to Field:
The manuscript entitled "Conserved Pib2 regions have distinct roles in TORC1 regulation at the vacuole" by Troutman et al., describes about regulatory mechanism of Pib2, which regulates TORC1, by making mutant and chimera proteins. The authors narrowed down the amino acids residues in Pib2 that suppressively functions in TORC1 regulation. They also found that FYVE domain is not necessary for recovery from rapamycin exposure. In addition, they described that endosomal localization of Pib2 is not needed for this process.

Reviewer 2 Comments for the Author:
In general, the data are mostly convincing and the experiments were moderately performed. However, regrettably, this study lacks enthusiastic novelty that warrant the publication in JCS. Most of novel points described in this study is detailed analysis of the preceding works but not leading to our deepened understanding of the mechanisms.

We thank the reviewer for their helpful suggestions.

Specific points
1 Figure 1 C. The authors concluded that rapamycin recovery is defected in pib2 null and several mutants based on plating assay. Does this mean that the cells are irreversibly dead? The authors need to support this point from the other line of evidence. What does rapamycin recovery means in mechanistic context. Without it, it is not enough for this journal.

In the context of the rapamycin recovery assays, we are assaying the ability of the cells to reactivate TORC1 to promote cell growth. With the rapamycin concentration being used (200 ng/ml) in this time frame, the cells are arrested in the G1 phase (Barbet et al, 1996).

© 2022. Published by The Company of Biologists under the terms of the Creative Commons Attribution License (https://creativecommons.org/licenses/by/4.0/).

To further confirm that the cells are not dead following rapamycin exposure, and remain viable, we used the stain FM4-64. FM4-64 is actively endocytosed and can therefore only stain the vacuoles of living cells (Vida and Emr, 1995). Here, we treated both W303A and Δ*pib2* cells with 200 ng/ml rapamycin for 4 hours, washed away the rapamycin, and allowed the cells to recover for 48 hours. At 48 hours, we stained the cells with FM4-64 for 1 hour, followed by a 1-hour wash with synthetic complete (SC) media. Both W303A and Δ*pib2* cells had stained vacuoles. This has also been previously shown (Varlakhanova et al, 2017).

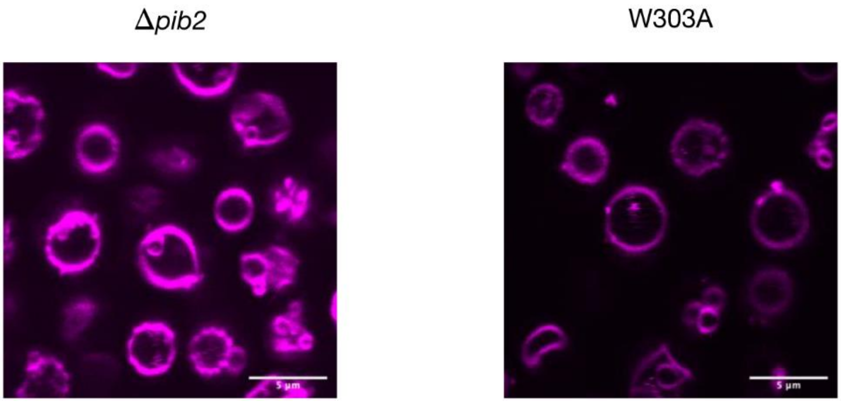

2 Figure 3. It deserved to be reported that specific amino acids necessary for TORC1 suppression. However the analysis were shallow for this journal. For example, does S to A mutant actually affect the phosphorylation status of Pib2?n Line 234 How about K to R mutant?
To address this, we have generated both phospho-dead (S→A) and phosphomimetic (S→E) mutants for the key serine residues in region B (S113 and S118). Pib2 S113A is known to be phosphorylated by Cdk1 (Holt et al., 2009) and we therefore would expect phosphorylation at this site to be affected by these mutations. Further, our rapamycin assay results showed distinct differences in growth between these phospho-dead and phosphomimetic mutants (Fig. 3B).

We have furthermore generated a 59KKK61→RRR mutant to address the K→R mutation in region A. This K→R mutant recovered just as WT Pib2 did, supporting that the positive charge is needed for inhibitory function (Fig. 3B).

3 Figure 6B The picture is missing superscript.
Thank you. The appropriate labels were added.

4 The conclusion obtained from chimera proteins are mostly similar to that of Hatakeyama paper.
The conclusions of the chimeric proteins do indeed support the conclusions of Hatakeyama et al., 2019. Hatakeyama et al, show that TORC1 activity at the vacuole promotes protein synthesis and cell growth via phosphorylation of Sch9, whereas endosomal TORC1 activity inhibits autophagy through phosphorylation of Atg13 and Vps27. Hatakeyama et al did not investigate the roles of TORC1 regulators at these different pools of TORC1. Our contribution here is to investigate the role of Pib2 in reactivation of TORC1 at these pools. We therefore targeted Pib2 to the vacuole or endosome and used cell growth assays as a readout of TORC1 activation. Our results showed that only vacuolar Pib2 was sufficient to reactivate TORC1 activity to promote cell growth. This agrees with and supports the results of Hatakeyama et al 2019 in that vacuolar TORC1 is needed to promote cell growth and highlights that Pib2 is an essential part of this process in terms of TORC1 reactivation. This is also addressed in the discussion ~L565. This localization dependent activity of Pib2 hints at the possibility that upstream TORC1 regulators and/or their functions may differ depending on localization. This challenges the paradigm that TORC1 regulators regulate all TORC1 functions.

© 2022. Published by The Company of Biologists under the terms of the Creative Commons Attribution License (https://creativecommons.org/licenses/by/4.0/).

Second decision letter

MS ID#: JOCES/2022/259994

MS TITLE: Conserved Pib2 regions have distinct roles in TORC1 regulation at the vacuole

AUTHORS: Kayla K Troutman, Natalia V Varlakhanova, Bryan A Tornabene, Rajesh Ramachandran, and Marijn Gerard Johannes Ford
ARTICLE TYPE: Research Article

I am happy to tell you that your manuscript has been accepted for publication in Journal of Cell Science, pending standard ethics checks.

Reviewer 1

Advance summary and potential significance to field

See my first review comments.

Comments for the author

The authors have addressed my points thoroughly and significantly improved the quality of their manuscript. In particular, the addition of EEA1-FIVE domain as a control enabled rigorous interpretation of their results on Pib2-FIVE domain in a comparative manner, significantly enhancing the value of these data. I would be totally happy with this version being published in JCS.*---

Reviewer 2

Advance summary and potential significance to field

The authors made detailed domain analysis of Pib2 and showed its function together with the significance of the localization on TORC1 regulation.

Comments for the author

Now the paper is acceptable.

© 2022. Published by The Company of Biologists under the terms of the Creative Commons Attribution License (https://creativecommons.org/licenses/by/4.0/).
